# KOM-SLAM: A GNN-Based Tightly Coupled SLAM and Multi-Object Tracking Framework

**DOI:** 10.3390/s26010128

**Published:** 2025-12-24

**Authors:** Jinze Liu, Ye Tian, Yanlei Gu, Shunsuke Kamijo

**Affiliations:** 1 Graduate School of Information Science and Technology, The University of Tokyo, Tokyo 113-0033, Japan; jzliu@umich.edu; 2 Institute of Industrial Science, The University of Tokyo, Tokyo 153-8505, Japan; 3 Graduate School of Advanced Science and Engineering, Hiroshima University, Hiroshima 739-8527, Japan; guyanlei@hiroshima-u.ac.jp

**Keywords:** SLAM, multi-object tracking, GNN

## Abstract

Coupled simultaneous localization and mapping (SLAM) and multi-object tracking have been studied in recent years. Although these tasks achieve promising results, they mainly associate keypoints and objects across frames separately, which limits their robustness in complex dynamic scenes. To overcome this limitation, we propose KOM-SLAM, a tightly coupled SLAM and multi-object tracking framework based on a Graph Neural Network (GNN), which jointly learns keypoint and object associations across frames while estimating ego-poses in a differentiable manner. The framework constructs a spatiotemporal graph over keypoints and object detections for association, and employs a multilayer perceptron (MLP) followed by a sigmoid activation that adaptively adjusts association thresholds based on ego-motion and spatial context. We apply a soft assignment on keypoints to ensure differentiable pose estimation, enabling the pose loss to supervise the association learning directly. Experiments on the KITTI Tracking demonstrate that our method achieves improved performance in both localization and object tracking.

## 1. Introduction

Simultaneous localization and mapping (SLAM) and multi-object tracking are two essential tasks for autonomous systems. SLAM estimates the ego-pose (the current position and orientation of the sensor/robot) and builds a map of the surrounding environment [1,2], while multi-object tracking tracks the motion of detected objects to support safe decision-making [3,4]. Although these tasks are traditionally developed independently, the two tasks are naturally complementary. SLAM provides accurate ego-pose estimates for tracking, while object tracking helps identify dynamic objects, improving SLAM robustness in dynamic environments.

Tightly coupled SLAM and multi-object tracking systems usually build a factor graph of odometry and object states, and a joint optimization is applied to improve the accuracy and robustness of localization and object tracking using temporal and spatial constraints [5,6]. However, most existing approaches rely on manually designed heuristics for data association, such as descriptor similarity for keypoints and spatial proximity for objects. These heuristic associations can be brittle in cluttered or ambiguous environments, particularly when multiple dynamic objects occlude or overlap. Additionally, treating keypoint and object associations independently prevents full exploitation of the shared information between feature extraction and object detection.

Graph Neural Networks (GNNs) have emerged as powerful tools for learning associations in structured data by leveraging message passing to aggregate information from connected neighbors [7,8]. While GNNs have been applied to tasks like keypoint matching and object tracking independently [9,10], they have not been fully explored in a tightly coupled SLAM and multi-object tracking framework, where keypoint association and object association could be learned jointly. Moreover, most existing GNN-based approaches are not designed to support differentiable pose estimation, limiting their integration into end-to-end learning pipelines.

In response to these challenges, we propose KOM-SLAM, a tightly coupled SLAM and multi-object tracking framework, which jointly matches keypoints and objects across frames with a GNN. Our approach builds a graph that includes both keypoints and object detections across frames, enabling the GNN to simultaneously find the association between keypoints and between objects. Furthermore, to improve robustness under varying motion patterns, we apply a Multilayer Perceptron (MLP)–Sigmoid gating layer to restrict the association dynamically based on ego-motion and the spatial distance of each keypoint from the ego-pose. In addition, we adopt a soft assignment scheme over the keypoint similarity matrix, which enables differentiable pose estimation and allows the final odometry loss to directly supervise the GNN association learning.

The main contributions of the new proposed GNN-based, tightly coupled SLAM and multi-object tracking system are the following:

1. We propose KOM-SLAM, a GNN-based, tightly coupled SLAM and multi-object tracking framework, where the GNN jointly learns associations between keypoints and objects. A soft assignment mechanism is applied to backpropagate the pose estimation loss through the GNN. To the best of our knowledge, this is the first learning-based system that tightly integrates SLAM and multi-object tracking.
2. We embed the ego motion and spatial distance between the keypoint and the ego pose in the network to allow the dynamic adjustment of the keypoint matching range.
3. We validate the effectiveness of KOM-SLAM on the KITTI Tracking dataset, demonstrating improved performance in both pose estimation and object tracking.

The rest of the paper is organized as follows. Section 2 reviews the related work. Section 3 presents the proposed GNN-based, tightly coupled SLAM and multi-object tracking framework in detail. Experimental results and evaluations are demonstrated in Section 4. Finally, Section 5 concludes the paper and discusses directions of future work.

## 2. Related Work

### 2.1. Coupled SLAM and Multi-Object Tracking

Traditional SLAM systems rely on feature extraction and frame-to-frame correspondence to estimate ego-motion, using corner keypoints in visual inputs [11] or edge and planar points in Light Detection and Ranging (LiDAR) data [12]. However, including keypoints on dynamic objects in pose estimation introduces errors due to unmodeled motion. To address this, dynamic observation methods have been proposed to filter out potentially dynamic points—either by removing features associated with detected objects [13,14] or by segmenting dynamic regions using photometric or geometric cues [15]. The robustness of these methods is heavily dependent on the quality of the road scene understanding. Recent benchmarks, such as RSUD20K [16], have advanced the state-of-the-art in road scene understanding by providing diverse and high-resolution data for object detection. While effective in avoiding dynamic noise, these approaches often lack the ability to model or track object motion, limiting the SLAM system’s robustness in dynamic environments.

To improve robustness in dynamic environments, loosely coupled systems combine SLAM and multi-object tracking, where each component operates independently but shares information to enhance overall performance. In these frameworks, object tracking typically helps identify and remove dynamic points from sensor data, while SLAM uses the filtered static features to perform accurate ego-pose estimation. MaskFusion [17] and DOT [18], the visual dynamic object tracking methods for SLAM, rely on reprojection or photometric errors to track object motion with object masks and identify static keypoints. Instead of tracking image masks, ClusterSLAM [19] and ClusterVO [20] project keypoints into 3D, track 3D clusters of points, and estimate camera poses based on static features. LiDAR-based methods take advantage of the accurate depth measurement and object detection, often using Kalman filtering and point cloud registration to track objects [21]. Hybrid LiDAR-visual systems, such as Dynam-LVIO [22], benefit from sensor redundancy and use both the iterative closest point (ICP) error and reprojection error to update a Kalman filter to track objects. While these loosely coupled designs are modular and flexible, their ego-poses estimation only relies on the static features, which may limit their effectiveness in highly dynamic scenes where static features are not sufficient.

In contrast to loosely coupled methods, tightly coupled SLAM and multi-object tracking frameworks typically jointly optimize ego-poses, object poses, and object motions with a factor graph in the backend. This joint optimization allows dynamic object observations and motion models to directly support ego-pose estimation, which is particularly beneficial when static features are sparse or occluded. Visual systems usually calculate reprojection errors as constraint functions in the joint optimization. DynaSLAM II [5] and CubeSLAM [23] detect 2D boxes and optimize object points with factor graphs, while segmentation-based approaches like TwistSLAM [24] apply optical flow to track the object points. Recent advances also explore richer representations, including quadrics [6] and articulated models for non-rigid bodies [25], improving robustness across object types. The LiDAR-based methods, such as DL-SLOT [26] and LIMOT [27], benefit from accurate 3D detections and pay more attention to the object trajectory prediction and association. Instead of applying constant motion constraints, LIO-LOT [28] applies a constant acceleration and angular velocity motion model for objects. IMM-SLAMMOT [29] includes multiple motion models in the factor graph to be more adaptive to the complex real-world dynamic scenarios. The switching-coupled systems combine the loosely coupled factors to avoid unreliable objects causing performance degradation [30,31]. Despite their effectiveness, most existing tightly coupled methods rely on heuristic-based association strategies, leaving room for learning-based techniques to better capture complex correspondence patterns.

### 2.2. GNN-Based Matching

Graph Neural Networks (GNNs) have emerged as a powerful tool for learning associations in structured data, such as object tracking and keypoint matching across frames. In multi-object tracking, Wang et al. use the 2D appearance feature as nodes, define the edges between the nodes within pixel distance thresholds across frames, and apply GraphConv as their aggregation method [32]. Similarly, PTP [33] connects edges based on 3D distance and applies GraphConv; however, it utilizes Long Short-Term Memory (LSTM) and MLP to encode 3D detection results into positioning features as GNN nodes. Instead of using GraphConv, GNN3DMOT [3] improves the tracking performance by applying an attention-based weight to aggregate the difference in features between nodes and neighborhood nodes. Brasó and Leal-Taixé build a graph with multiple frames and design a time-aware message-passing network to update the node features [34]. Following it, SUSHI [10] and Bilgi et al. [35] perform detection association over different hierarchies of timespans to improve long-term association. At each hierarchical level, a shared-weights GNN is applied. Chen et al. apply GNN to multi-object tracking in satellite videos and focus on the tiny object tracking task [36]. Instead of using heuristic methods to define GNN edges, Gao et al. design NodeNet to generate the edge connections for GNN, constructed by an encoder and a decoder [37].

In keypoint matching, SuperGlue [9] represents keypoints as graph nodes with both descriptor and positioning features. It uses alternating self-attention layers, where nodes in the same frame are fully connected, and cross-attention layers, where nodes are fully connected across frames. Following SuperGlue, LightGlue [38] uses the positioning information to calculate the attention scores in self-attention layers and uses descriptor similarity in cross-attention layers. To improve the time efficiency of SuperGlue, SGMNet [39] applies a seeding module to generate a small set of matches as seeds. Subsequently, a Seeded GNN is applied to utilize seed matches to pass messages across frames and within frames through attentional aggregation. Similarly, ClusterGNN [40] reduces the number of edges by operating GNN on clusters of keypoints. They use the K-means algorithm to cluster the query and key vectors, and then apply a cluster-based sparse attention to aggregate the features. Furthermore, LoFTR [41] combines the GNN with a local feature detector to build an end-to-end trainable model. It enables the matching model to supervise the local feature convolutional neural network (CNN), which outperforms using a pre-built keypoint detector. These works collectively demonstrate GNNs’ strength in capturing contextual cues and finding correspondence in matching tasks. However, most existing works perform the object association and keypoint association independently, and the GNN approaches are not fully explored in the coupled object and keypoint matching.

## 3. Method

### 3.1. System Architecture

**Figure 1:**
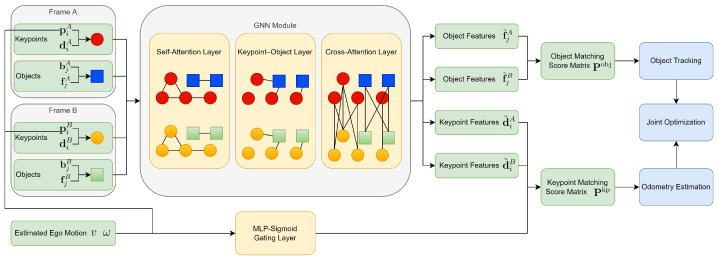
Overall architecture of KOM-SLAM. The figure illustrates the tightly coupled Graph Neural Network (GNN) framework. It shows the flow from keypoints and object inputs through the GNN association module to the joint optimization backend for estimating ego-pose and object states.

The overall structure of the proposed GNN-based coupled SLAM and multi-object tracking system is illustrated in Figure 1. The system takes the extracted keypoints and object detections from two frames of perception data at timestamps *A* and *B* as input. Its objective is to establish associations between keypoints and between objects across frames and estimate the relative transformation $T_{A}^{B}\in \mathrm{SE}\left(3\right)$, which maps coordinates from frame *A* to frame *B*. In practice, we use frames *A* and *B* that are temporally adjacent (i.e., consecutive frames), as this ensures sufficient spatial overlap and stable geometric consistency for both keypoint and object associations. The framework does not strictly require consecutive frames, but larger temporal gaps generally increase motion magnitude and reduce association reliability. The impact of reduced frame density and increased time intervals is empirically evaluated in Section 4.4.3.

Each keypoint *i* in frame t∈{A,B} is represented as a tuple $\left(p_{i}^{t},d_{i}^{t}\right)$, where $p_{i}^{t}\in R^{3}$ denotes the 3D position, and $d_{i}^{t}\in R^{F_{k}}$ is the corresponding descriptor. Similarly, each object detection *j* in frame *t* is defined as $\left(b_{j}^{t},f_{j}^{t}\right)$, where $b_{j}^{t}=\left(x_{j},y_{j},z_{j},l_{j},w_{j},h_{j},\theta_{j}\right)$ denotes the 3D bounding box parameters (position, size, and yaw angle) and $f_{j}^{t}\in R^{F_{o}}$ is the associated appearance feature.

In practice, the input data $\left(p_{i}^{t},d_{i}^{t}\right)$ and $\left(b_{j}^{t},f_{j}^{t}\right)$ are derived from stereo image inputs. Specifically, 2D keypoints and their descriptors are extracted from left images using SuperPoint [11], and their 3D coordinates $p_{i}^{t}$ are estimated by triangulation using depth inferred from stereo images and camera intrinsic matrix. Object detections are generated by PointRCNN [42], which provides 3D bounding box parameters $b_{j}^{t}$. The associated appearance features $f_{j}^{t}$ are extracted by applying a pretrained ResNet backbone to the cropped image region of each detected object.

The features and positioning information of keypoint and object are encoded as nodes in an attention-based GNN, including self-attention, keypoint–object, and cross-attention layers. In the GNN layers, the positioning attributes remain fixed and are used to guide the attention mechanism, while the feature vectors are the only components updated. The updated features are then used to compute matching score matrices across frames. For keypoints, an auxiliary matrix is additionally introduced to guide the similarity computation by incorporating the estimated relative transformation and spatial distances between the keypoints and the ego-poses.

Let $N_{A}$ and $N_{B}$ denote the number of keypoints extracted in timestamps *A* and *B*, respectively. Soft assignment is applied to the keypoint matching score matrix $P^{\mathrm{kp}}\in R^{N_{A}\times N_{B}}$ to obtain the corresponding keypoints $\left\{p_{k}^{A},p_{k}^{B}\right\}$. These correspondences are then used to estimate the relative transformation $T_{A}^{B}$ via a differentiable optimization module. For object associations, a set of object correspondences $\left(i,j\right)\subset M_{A}\times M_{B}$ is established through the object matching score matrix $P^{\mathrm{obj}}\in R^{M_{A}\times M_{B}}$, where $M_{A}$ and $M_{B}$ are the number of detected objects in frames *A* and *B*, respectively. Unlike keypoints, soft assignment is not applied to objects due to their discrete and non-repeatable nature.

Finally, ego-pose optimization is performed by minimizing the alignment error of the matched keypoints. To further refine both pose and object trajectories, a factor graph is constructed over a sliding window to jointly optimize relative transformations, object poses, and object motions. The use of soft associations for keypoints, together with differentiable optimization, ensures that the entire framework is trainable.

### 3.2. Graph Neural Network

**Figure 2:**
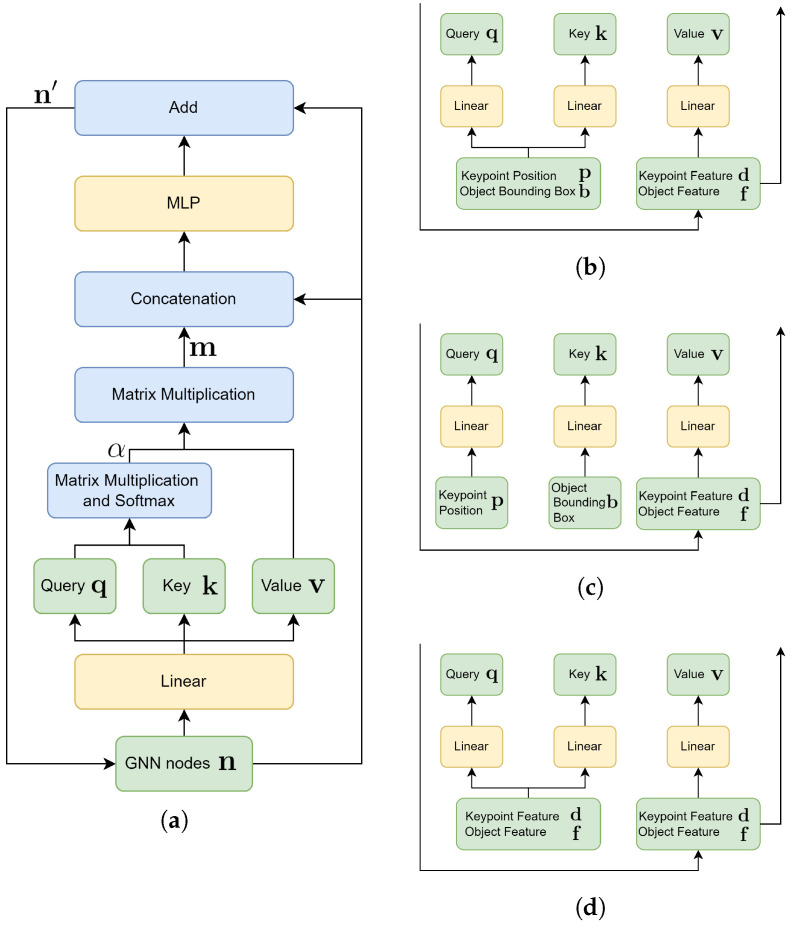
(**a**) Overall architecture of the proposed GNN attention module. (**b**) Query, key, and value generation in the self-attention layer for intra-entity, intra-frame feature refinement. (**c**) Query, key, and value generation in the keypoint–object layer for cross-entity, intra-frame message passing between keypoints and objects. (**d**) Query, key, and value generation in the cross-attention layer for intra-entity, inter-frame feature exchange.

As illustrated in Figure 2, the proposed attention-based GNN jointly processes keypoints and object detections from two frames by propagating and refining node features through geometric and appearance-based relationships both within and across timestamps.

Each graph node is represented as a pair n=(x,g), where x denotes the learnable feature vector (keypoint descriptor $d_{i}$ or object appearance feature f), and g denotes the corresponding positional attribute (keypoints 3D position p or object bounding box parameters b). While g remains fixed throughout message passing, it is used in the attention mechanism to construct queries and keys, thereby guiding how information flows between nodes. Only the feature x is updated layer by layer.

GNN edges are constructed differently across three layers to capture complementary contextual relationships. In the self-attention layer, keypoint nodes are connected to other keypoints within a predefined distance threshold in the same frame. Similarly, object nodes are connected to nearby object nodes in the same frame. This layer captures local spatial relationships. In the keypoint–object layer, the keypoint nodes and object nodes in the same frame are connected if a keypoint lies inside the 3D bounding box of the object, allowing keypoints to incorporate higher-level object context. Lastly, in the cross-attention layer, keypoints are connected to keypoints in the other frame within a spatial threshold. Likewise, object nodes are connected across frames based on spatial distance. This layer facilitates temporal correspondence reasoning across frames.

At each layer of GNN, node features are updated through attention-based message passing from its connected neighbors. Specifically, the updated feature $x_{i}^{\prime }$ for node *i* is calculated by combining the original feature $x_{i}$ with the aggregated message $m_{i}$ via a 2-layer MLP, as defined in Equation (1):

(1) $$x_{i}^{\prime }=x_{i}+\mathrm{MLP}\left(\left[x_{i}∥m_{i}\right]\right),$$

where $n_{i}^{\prime }=\left(x_{i}^{\prime },g_{i}\right)$ is the updated GNN node, and ∥ denotes concatenation operation. The 2-layer architecture is chosen to provide sufficient expressive power for feature refinement while maintaining computational efficiency and preventing over-fitting.

The aggregated message $m_{i}$ is defined as a weighted sum over neighbors N(i), calculated by Equations (2) and (3):

(2) $$\begin{array}{cc} m_{i} & =\sum_{j\in N(i)}\alpha_{ij}v_{j}, \end{array}$$

(3) $$\begin{array}{cc} \alpha_{ij} & ={\mathrm{softmax}}_{k}{\left(q_{i}^{T}k_{k}\right)}_{j}, \end{array}$$

where v, q, and k denote the value, query, and key vectors, respectively. These vectors are obtained by applying different linear layers to the node features. The specific choice of input features depends on the type of layer, as shown in Figure 2. In the self-attention layers, keypoints embed positioning information for queries and keys, while their descriptors are applied to generate value vectors. Object nodes follow the same pattern, using bounding boxes to generate queries and keys. In the keypoint–object layers, queries are derived from keypoint positions and keys from object bounding boxes, while the values are taken from either keypoint descriptors or object features, enabling information exchange between keypoints and objects. Finally, in the cross-attention layers, queries, keys, and values are all constructed from descriptors (for keypoints) or appearance features (for objects), so that message passing emphasizes similarity in feature space across frames.

Each GNN layer is applied sequentially, and multiple iterations of message passing can be performed to allow contextual information to propagate throughout the graph. The updated keypoint and object features are then used for computing similarity matrices and downstream estimation tasks.

### 3.3. Matching Score Matrix Calculation

To determine correspondences between keypoints and between objects across two frames *A* and *B*, we compute two matching score matrices for keypoints and objects based on the updated node features obtained from the GNN. Since the processes are similar, we describe the calculation for keypoints, while the object case follows identically.

Let $D^{A}\in R^{N_{A}\times F_{k}}$ and $D^{B}\in R^{N_{B}\times F_{k}}$ denote the updated descriptors matrices for keypoints in frames *A* and *B*, respectively, where each row corresponds to $D_{i\cdot }^{t}:={\tilde{d}}_{i}^{t}$, the updated features of keypoint *i* in frame *t*. We first calculate a similarity matrix $S\in R^{N_{A}\times N_{B}}$ using the inner product of the feature matrices, as shown in Equation (4):

(4) $$S=D^{A}{\left(D^{B}\right)}^{⊤}.$$

We define the raw matching score matrix $P^{\mathrm{raw}}\in R^{N_{A}\times N_{B}}$ as the sum of the row-wise and column-wise softmax normalizations of S in Equation (5):

(5) $$P_{ij}^{\mathrm{raw}}={\mathrm{softmax}}_{k}{\left(S_{ik}\right)}_{j}+{\mathrm{softmax}}_{k}{\left(S_{kj}\right)}_{i}.$$

In addition to descriptor similarity, we introduce an auxiliary matrix $P^{\mathrm{aux}}\in {\left[0,1\right]}^{N_{A}\times N_{B}}$ based on keypoint positions and estimated ego-motion. As shown in Equation (6), we compute a learned threshold $\tau_{ij}$ using a lightweight network, which estimates the possible range that the corresponding keypoint might be in for each keypoint:

(6) $$\tau_{ij}=\mathrm{MLP}\left([{∥p_{i}^{A}∥}_{2}∥v_{\mathrm{ego}}∥\omega_{\mathrm{ego}}]\right),$$

where ${∥p_{i}^{A}∥}_{2}$ is the Euclidean distance from keypoint *i* to the ego pose in frame *A*, and $v_{\mathrm{ego}}$ and $\omega_{\mathrm{ego}}$ represent the estimated translational and rotational velocities of ego-motion between frames *A* and *B*. The auxiliary matrix, which acts as a learned spatial prior, is then defined using the sigmoid function σ in Equation (7):

(7) $$P_{ij}^{\mathrm{aux}}=\sigma \left(\tau_{ij}-\delta_{ij}\right),$$

where $\delta_{ij}={∥p_{i}^{A}-p_{j}^{B}∥}_{2}$ is defined as the distance between the keypoint pair (i,j).

Finally, the keypoint matching score matrix $P\in R^{N_{A}\times N_{B}}$ is obtained by element-wise multiplication of the raw matching score matrix $P^{\mathrm{raw}}$ and the auxiliary matrix $P^{\mathrm{aux}}$, as shown in Equation (8):

(8) $$P=P^{\mathrm{raw}}⊙P^{\mathrm{aux}}.$$

This fused matrix P is used for soft keypoint matching and subsequent relative pose estimation. Note that for objects, the raw matching score matrix is directly used as the matching score matrix.

### 3.4. Correspondence Association and Joint Optimization

Given the matching score matrices computed from the updated keypoint and object features, our framework identifies inter-frame correspondences and jointly optimizes the relative ego-motion and object states.

Object correspondences are first determined through an argmax operation combined with a mutual consistency check. Let $P^{\mathrm{obj}}\in R^{M_{A}\times M_{B}}$ denote the object-level matching score matrix between the $M_{A}$ and $M_{B}$ detected objects in frames *A* and *B*, respectively. To ensure that both objects select each other as the most similar match, a candidate correspondence (i,j) is selected if both conditions in Equations (9) and (10) are met:

(9) $$\begin{array}{cc} j & ={\mathrm{argmax}}_{k}P_{ik}^{\mathrm{obj}}, \end{array}$$

(10) $$\begin{array}{cc} i & ={\mathrm{argmax}}_{k}P_{kj}^{\mathrm{obj}}. \end{array}$$

Keypoint correspondences are derived using soft assignment. Given the keypoint score matrix $P^{\mathrm{kp}}\in R^{N_{A}\times N_{B}}$, the corresponding location in frame *B* for each keypoint $pi^{A}$ in frame *A* is estimated as a weighted average, as defined in Equation (11):

(11) $${\hat{p}}_{i}^{B}=\sum_{j=1}^{N_{B}}\frac{\exp(P_{ij})}{\sum_{k=1}^{N_{B}}\exp\left(P_{ik}\right)}\cdot p_{j}^{B}.$$

Crucially, this soft assignment mechanism is designed to maintain differentiability, allowing the matching scores to be directly supervised by the downstream odometry loss.

The resulting set of soft correspondences $\left(p_{i}^{A},{\hat{p}}_{i}^{B}\right)$ is then used to estimate the relative transformation $T_{A}^{B}\in \mathrm{SE}\left(3\right)$ by minimizing the sum of squared alignment errors $E_{\mathrm{rel}}$ defined in Equation (12):

(12) $$E_{\mathrm{rel}}=\sum_{i}{∥{\hat{p}}_{i}^{B}-T_{A}^{B}\cdot p_{i}^{A}∥}_{2}^{2}.$$

This optimization is solved using the Levenberg–Marquardt (LM) algorithm [43], implemented in a differentiable form with Theseus [44]. This transformation aligns keypoints from frame *A* to frame *B* and provides an initial estimate of ego-motion. For the initial guess of $T_{A}^{B}$ in the optimization, we use a constant motion model. For the first frame, $T_{A}^{B}$ is initialized as an identity transformation.

To further refine the ego-motion estimate and incorporate object dynamics, we employ a joint optimization framework with a factor graph following [27]. The graph consists of ego-pose nodes, object pose nodes, and object motion nodes spanning a fixed-length sliding window (selected to be 5) of recent frames. Let X denote the set of all variables in the graph. The following factors are defined: (i) odometry constraints $E_{\mathrm{odom}}$ between consecutive ego-poses; (ii) observation constraints $E_{\mathrm{obs}}$ linking ego-poses to observed object poses; (iii) motion constraints $E_{\mathrm{motion}}$ connecting consecutive object poses and motion; and (iv) constant velocity constraints $E_{\mathrm{const}}$ between object motion nodes, as shown in Equation (13):

(13) $$X^{*}=\underset{X}{\mathrm{argmin}}\left(E_{\mathrm{odom}}+E_{\mathrm{obs}}+E_{\mathrm{motion}}+E_{\mathrm{const}}\right),$$

where $X^{*}$ denotes the optimized factor graph nodes. The optimization is again performed using the LM algorithm [43] with Theseus [44], maintaining full differentiability. This joint optimization allows for mutual refinement of ego-motion and object tracking, enhancing both consistency and accuracy. Crucially, both the soft assignment mechanism and the factor graph optimization are implemented in a differentiable manner, forming the foundation for our learning pipeline.

### 3.5. Training Loss

To supervise the learning process of KOM-SLAM, we design a composite loss function that supervises object association, keypoint correspondence estimation, and ego-motion prediction. The overall loss is defined in Equation (14):

(14) $$L=\lambda_{\mathrm{obj}}L_{\mathrm{obj}}+\lambda_{\mathrm{kp}}L_{\mathrm{kp}}+\lambda_{\mathrm{odom}}L_{\mathrm{odom}},$$

where $\lambda_{\mathrm{obj}}$, $\lambda_{\mathrm{kp}}$, and $\lambda_{\mathrm{odom}}$ are weighting coefficients.

Ground-truth tracking IDs are used to establish object correspondences between frames *A* and *B*. Let ${\hat{P}}^{\mathrm{obj}}$ denote the ground truth object matching score matrix, where ${\hat{P}}_{ij}^{\mathrm{obj}}=1$ for the objects with the same tracking IDs, ${\hat{P}}_{ij}^{\mathrm{obj}}=0$ for objects with different tracking IDs. Following PTP [33], we apply the BCE loss for each entry of $P^{\mathrm{obj}}$ and the CE loss for each column of $P^{\mathrm{obj}}$, as shown in Equation (15):

(15) $$L_{\mathrm{obj}}=L_{\mathrm{BCE}}+L_{\mathrm{CE}}.$$

Ground truth matched keypoint pairs are identified using both spatial distance and descriptor similarity after aligning keypoints in frame *A* to the coordinate frame of *B* using the ground-truth relative transformation $T_{A}^{B,\mathrm{gt}}$. A pair (i,j) is considered positive if the spatial distance (Equation (16)) and descriptor similarity (Equation (17)) criteria are met:

(16) $$\begin{array}{cc} ∥T_{A}^{B,\mathrm{gt}}\cdot p_{i}^{A}-p_{j}^{B}{∥}_{2} & <\delta_{\mathrm{gt}}, \end{array}$$

(17) $$\begin{array}{cc} \cos(d_{i}^{A},d_{j}^{B}) & >\delta_{\mathrm{sim}}, \end{array}$$

where $\delta_{\mathrm{dist}}$ and $\delta_{\mathrm{sim}}$ are distance and descriptor similarity thresholds, respectively. Let $P_{\mathrm{gt}}^{\mathrm{kp}}$ denote the sets of ground truth matched keypoint pairs. As shown in Equation (18), following LightGlue [38], the keypoint matching loss is formulated as mean negative log-likelihood over the ground truth matched pairs:

(18) $$L_{\mathrm{kp}}=-\frac{1}{|P_{\mathrm{gt}}^{\mathrm{kp}}|}\sum_{\left(i,j\right)\in P_{\mathrm{gt}}^{\mathrm{kp}}}\log P_{ij}^{\mathrm{kp}}.$$

To supervise ego-motion estimation, we penalize both translation and rotation errors between the predicted transformation $T_{A}^{B}$ and the ground-truth $T_{A}^{B,\mathrm{gt}}$. Let $q_{\mathrm{est}},q_{\mathrm{gt}}\in R^{4}$ denote the estimated and ground-truth unit quaternions, and $t_{\mathrm{est}},t_{\mathrm{gt}}\in R^{3}$ denote the translation vectors. The transformation loss, $L_{\mathrm{odom}}$, is defined as the sum of the rotation and translation errors, as shown in Equation (19):

(19) $$L_{\mathrm{odom}}=\beta \cdot {∥q_{\mathrm{est}}-q_{\mathrm{gt}}∥}_{2}+{∥t_{\mathrm{est}}-t_{\mathrm{gt}}∥}_{2},$$

where β is a weighting factor to balance the contributions of rotational and translational errors. The factor β is introduced for scale normalization, which is necessary because the rotational error (distance between unit quaternions) and the translational error (in meters) typically have different magnitudes. This ensures that both components contribute equitably to the overall loss gradient during training.

## 4. Experiments

We conduct experiments on the KITTI Tracking dataset [45] to evaluate the effectiveness of KOM-SLAM. Section 4.1 describes the implementation details and dataset setup. The experimental evaluation is organized into two main parts: Section 4.2 presents the results for odometry estimation, while Section 4.3 evaluates the multi-object tracking performance.

### 4.1. Experimental Details

**Figure 3:**
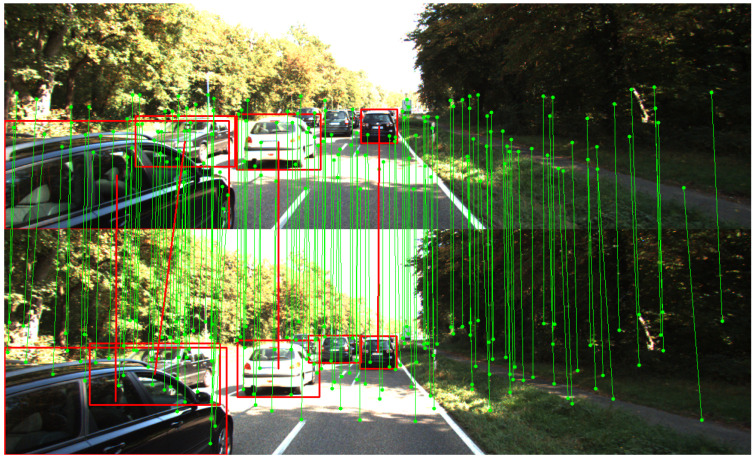
Keypoint and object correspondence example of our proposed framework on the KITTI tracking dataset. The green dots and the lines demonstrate the matched keypoints and their correspondence. The red rectangles and red lines represent the detected objects and their association.

For keypoint extraction, we apply SuperPoint [11], a self-supervised keypoint detector and descriptor, to generate keypoints from the images of the KITTI Tracking dataset. To obtain 3D object detections, we adopt PointRCNN [42], a two-stage 3D object detector that operates on LiDAR point clouds. For both models, we choose to use the pretrained weights available online. To fully evaluate KOM-SLAM on all the sequences of the KITTI Tracking dataset, we train two models. One is trained using sequences 0000–0010, while the other is trained with 0011, 0013, 0014, 0018, 0019, and 0020. This separation is necessary because we avoid evaluating a sequence using a model that has been trained on it. By training two models on complementary subsets, we ensure that every sequence can be used for evaluation without overlapping with its training data. Following prior works [5,24], the sequences 0012, 0015, 0016, and 0017 are excluded from the evaluation because they contain little or no camera motion. All models are trained using the Adam optimizer with a learning rate of $1\times 10^{-4}$ for 80 epochs and a batch size of 1. The GNN module consists of two layers, each integrating self-attention, cross-attention, and keypoint–object interaction mechanisms, with a feature dimension of 256. The training loss is a weighted combination of object association loss, keypoint association loss, and odometry consistency loss, with weights set to $\lambda_{\mathrm{obj}}=0.8$, $\lambda_{\mathrm{kp}}=1.0$, and $\lambda_{\mathrm{odom}}=0.6$, respectively. The overall balance factor is set to β=200.0. These hyperparameters were determined through a grid search. We observed that the system maintains stable performance within a ±10% range of the specified λ values. However, we found that reducing the factor β causes an increase in rotation error, because rotational errors are numerically smaller than translational errors. The proposed framework runs at approximately 49 ms per frame, where the GNN module takes approximately 22 ms per frame, and the backend optimization costs approximately 24 ms per frame. All experiments are conducted on a desktop system equipped with an NVIDIA GeForce RTX 4070 GPU. Figure 3 illustrates an example of the joint keypoint and object association results generated by KOM-SLAM on the KITTI Tracking dataset, highlighting successful correspondence in a challenging dynamic scene.

### 4.2. Odometry Estimation

This subsection presents the evaluation of ego-pose estimation performance using KOM-SLAM on the KITTI Tracking dataset. Specifically, sequences 0000–0010 are evaluated using a model trained on sequences 0011, 0013, 0014, 0018, 0019, and 0020, while the remaining sequences are tested using the model trained on 0000–0010. This split ensures that the test sequences are not seen during training, enabling a fair evaluation of generalization performance. We use the Relative Pose Error (RPE) as the evaluation metric to assess the accuracy of the estimated ego motion. RPE measures the local accuracy of the transformation between two consecutive frames and is particularly suitable for evaluating methods that emphasize pairwise frame association, such as ours.

Table 1 compares our method with three established baselines: ORB-SLAM2 [1], DynaSLAM [46], and DynaSLAM2 [5]. KOM-SLAM outperforms the baselines in both translational (RPE*_t_*) and rotational (RPE*_R_*) components on the majority of sequences. Notably, it achieves the lowest mean RPE*_t_* of 0.041 m/frame and the lowest mean RPE*_R_* of 0.028°/frame across all test sequences. This demonstrates the effectiveness of incorporating both keypoint- and object-level associations for robust ego-motion estimation in dynamic environments. Additionally, KOM-SLAM achieves the lowest standard deviation in translation and a standard deviation in rotation comparable to the best-performing method (0.020 vs. 0.015 of DynaSLAM [46]), demonstrating stable and consistent pose estimates across different sequences.

### 4.3. Multi-Object Tracking

This subsection presents the evaluation of multi-object tracking performance using KOM-SLAM on the KITTI Tracking dataset. We follow the evaluation of DynaSLAM2 [5], and compare object pose estimation accuracy across 12 longest object trajectories of the KITTI tracking dataset. We adopt standard evaluation metrics to assess the tracking performance: True Positives (TP) and Multi-Object Tracking Precision (MOTP), computed in 2D image space, Bird’s-Eye View (BV), and full 3D space. TP reflects the percentage of correctly tracked frames with respect to ground-truth annotations, while MOTP measures the average spatial alignment error of matched objects. Higher TP and MOTP indicate better tracking performance and localization accuracy. Table 2 summarizes the comparison between KOM-SLAM and DynaSLAM2. KOM-SLAM achieves higher TP and MOTP across all three modalities—2D, BV, and 3D—demonstrating substantial improvements in both tracking continuity and pose accuracy.

### 4.4. Ablation Study

To systematically analyze the contribution of each key component in KOM-SLAM, we conduct an ablation study focusing on the keypoint–object layer, attention-based GNN, the MLP–Sigmoid gating layer, the keypoint soft assignment mechanism, and the robustness under varying keypoint densities and temporal spacing.

#### 4.4.1. Ego Pose Estimation Ablation

Table 3 demonstrates the ego-pose estimation results for several ablated variants on the KITTI Tracking dataset. Specifically, we evaluate the following: (1) No Keypoint–Object Layer, where the keypoint–object interaction layer in the GNN is removed, and only the self-attention and cross-attention layers are used; (2) No GNN, where the attention-based GNN is removed, and associations are computed directly from raw keypoint features; (3) No Gating Layer, where the MLP–Sigmoid gating mechanism is disabled, and association scores are determined with the raw matching score matrix $P^{\mathrm{raw}}$; (4) No Soft Assignment, where hard assignment based on matching score matrix is used instead of soft assignment; and (5) KOM-SLAM, which corresponds to the full KOM-SLAM framework.

Across most sequences, the translational error (RPE*_t_*) remains relatively stable among different variants, indicating that coarse geometric alignment can still be obtained without learned association refinement. In contrast, the rotational error (RPE*_R_*) exhibits a clear sensitivity to the association strategy. In particular, removing the keypoint–object layer leads to noticeably higher rotation errors and variance, highlighting its importance for stable and accurate pose estimation.

The full model consistently achieves the lowest mean rotation error and standard deviation, confirming that the combination of keypoint–object interaction, GNN-based feature refinement, MLP–Sigmoid gating, and differentiable soft assignment is essential for robust ego-motion estimation. These results validate the necessity of our core design choices rather than relying on any single component alone.

#### 4.4.2. Object Tracking Association Ablation

To evaluate the effectiveness of the GNN-based association module for object tracking, we compare three variants using standard Multi-Object Tracking metrics: Multi-Object Tracking Accuracy (MOTA) and ID F1 Score (IDF1), as shown in Table 4. The compared methods include the following: (1) a Naive Approach, which associates objects solely based on the minimum Euclidean distance between the bounding box centers of the current detection and the predicted objects from the previous information; (2) No GNN, which uses raw appearance features without GNN-based refinement; (3) No Keypoint–Object Layer, which removes the keypoint–object interaction layer in GNN and only uses self-attention and cross-attention layers; and (4) KOM-SLAM, the full KOM-SLAM association pipeline.

For this ablation, ground-truth object detections are used to isolate the influence of the association mechanism and eliminate variance introduced by the upstream 3D detector. The naive distance-based approach performs poorly in sequences with frequent occlusions and interacting objects, leading to low MOTA and IDF1 scores. No GNN variant and no keypoint–object layer variant improve performance in most sequences by leveraging appearance cues, but still struggle with identity consistency under complex interactions.

The full model consistently achieves the highest MOTA and IDF1 scores in most sequences, demonstrating that the GNN architecture effectively captures relationships between objects. This results in more robust identity preservation and significantly improved tracking precision compared to heuristic or association strategies based on raw appearance feature matching.

#### 4.4.3. Data Density and Time Gap Analysis

To assess the robustness of KOM-SLAM under practical sensing constraints, we further analyze the impact of reduced keypoint density and increased temporal spacing between frames, with results summarized in Table 5. For the Sparser Keypoints variant, 50% of the detected keypoints are randomly retained in each frame. For the Every 2 Frames variant, only every second frame in each sequence is used for pose estimation.

When keypoint density is reduced, KOM-SLAM maintains comparable mean and standard deviation of the relative pose error, demonstrating strong robustness to sparse visual features. This indicates that the learned association mechanism can effectively find correspondences even with limited observations.

In contrast, increasing the temporal gap between frames results in noticeable degradation, particularly in rotation error. This effect is most pronounced in sequences with sharp turns (e.g., 0000, 0004, 0007), where larger inter-frame motion violates the small-motion assumption implicitly used in pairwise matching. These results highlight the importance of temporal resolution for accurate motion estimation, while also demonstrating that KOM-SLAM degrades gracefully under challenging conditions rather than failing catastrophically in sequences without large inter-frame motion.

## 5. Conclusions and Future Work

In this work, we introduced KOM-SLAM, a GNN-based framework that tightly couples odometry estimation and multi-object tracking. Our method constructs a spatiotemporal graph over keypoints and objects across frames, enabling the GNN to jointly update their features for robust association. A differentiable soft assignment mechanism is integrated with odometry estimation, allowing pose loss to directly supervise the learning process. To further enhance the robustness of keypoint association, we incorporate ego-motion and spatial priors into the matching process through an MLP–Sigmoid gating layer. We evaluated KOM-SLAM on the KITTI Tracking dataset, where it consistently outperformed strong baselines. The results demonstrate notable gains in both odometry accuracy and multi-object tracking precision, particularly in challenging sequences with frequent object motion and occlusion.

While KOM-SLAM demonstrates improvements in ego-motion estimation and multi-object tracking, the current framework presents several limitations that motivate future work.

Firstly, the system relies on high-quality external perception modules (SuperPoint for keypoints and PointRCNN for 3D detection). This dependency means the system’s overall performance is bounded by the accuracy and completeness of these detectors. Our learning pipeline currently mainly supervises the association, leaving the feature extraction and detection unsupervised.

Additionally, the system relies on frame-independent object detection and feature extraction, which can suffer from temporal inconsistency in bounding box parameters. While the inherent redundancy of keypoints provides robustness to this inconsistency in ego-pose estimation, the detection inconsistency severely challenges the factor graph optimization, as the constant velocity constraints applied to dynamic objects struggle to reconcile the detection noise with the smooth motion model, thereby risking the degradation of object trajectory accuracy and potentially destabilizing the coupled ego-pose estimate.

Furthermore, the system’s reliance on consecutive frame matching and a constant motion model for initialization makes the method sensitive to large motion changes. As demonstrated in our ablation study (matching every 2 frames), extremely high-speed scenarios or sequences with significant rotational velocity changes between frames can degrade performance, as the geometric prediction becomes unreliable.

Finally, while we demonstrate the excellent RPE performance, the current framework lacks long-term robustness mechanisms such as global mapping consistency and loop closure. The integration of GNN-based re-localization is necessary to prevent long-term drift in the ego-pose and object trajectories.

Building upon these limitations, future efforts will concentrate on three main directions: (1) integrating the front-end keypoint extraction and temporally consistent object detection modules into the differentiable pipeline for a truly end-to-end trainable system. (2) Exploring extensions to global consistency and long-term tracking, potentially by introducing map nodes into the GNN and designing a global loop closure factor for the optimization backend. (3) Expanding the evaluation and robustness analysis by applying KOM-SLAM to other diverse and challenging datasets, such as nuScenes [47] or Waymo [48], and also comparing with additional state-of-the-art visual odometry methods, such as DROID-SLAM [49] and DeepV2D [50]. 

## Figures and Tables

**Table 1:** Ego-pose estimation results on the KITTI Tracking dataset.

seq	ORB-SLAM2 [1]		DynaSLAM [46]		DynaSLAM2 [5]		KOM-SLAM	
	RPE_*t*_ (m/f)	RPE_*R*_ (°/f)	RPE_*t*_ (m/f)	RPE_*R*_ (°/f)	RPE_*t*_ (m/f)	RPE_*R*_ (°/f)	RPE_*t*_ (m/f)	RPE_*R*_ (°/f)
0000	**0.04**	**0.06**	**0.04**	**0.06**	**0.04**	**0.06**	**0.04**	0.07
0001	0.05	0.04	0.05	0.04	0.05	0.04	**0.04**	**0.03**
0002	0.04	0.03	0.04	0.03	0.04	0.02	**0.03**	**0.00**
0003	0.07	0.04	0.07	0.04	0.06	0.04	**0.05**	**0.02**
0004	0.07	**0.06**	0.07	**0.06**	0.07	**0.06**	**0.06**	0.07
0005	0.06	0.03	0.06	0.03	0.06	0.03	**0.05**	**0.01**
0006	0.02	0.04	0.02	0.04	0.02	**0.01**	**0.01**	**0.01**
0007	0.05	0.07	0.05	0.07	0.05	0.07	**0.04**	**0.03**
0008	0.08	0.04	0.08	0.04	0.10	0.04	**0.07**	**0.03**
0009	0.06	0.05	0.06	0.05	0.06	0.06	**0.04**	**0.02**
0010	0.07	0.04	0.07	0.04	0.07	0.03	**0.06**	**0.02**
0011	0.04	**0.03**	0.04	**0.03**	0.04	**0.03**	**0.03**	0.04
0013	0.04	0.05	0.04	0.05	0.04	0.04	**0.03**	**0.02**
0014	**0.03**	0.08	**0.03**	0.08	**0.03**	0.08	**0.03**	**0.05**
0018	0.05	0.03	0.05	0.03	0.05	0.02	**0.04**	**0.00**
0019	0.05	0.03	0.05	0.03	0.05	**0.02**	**0.03**	**0.02**
0020	0.11	0.07	0.05	0.04	0.07	0.04	**0.04**	**0.01**
mean	0.055	0.046	0.051	0.045	0.053	0.041	**0.041**	**0.028**
std	0.021	0.016	0.015	**0.015**	0.018	0.019	**0.014**	0.020

Bold values indicate the best performance for each metric in the corresponding row.

**Table 2:** Object pose estimation comparison on KITTI tracking dataset.

seq/obj.id/class	DynaSLAM2 [5]						KOM-SLAM					
	2D TP	2D MOTP	BV TP	BV MOTP	3D TP	3D MOTP	2D TP	2D MOTP	BV TP	BV MOTP	3D TP	3D MOTP
03/1/car	50.00	71.79	39.34	56.61	38.53	48.20	**95.90**	**86.85**	**95.90**	**75.52**	**97.54**	**66.19**
05/31/car	28.96	60.30	14.48	46.84	11.45	34.20	**82.09**	**83.63**	**81.42**	**76.97**	**83.78**	**66.15**
10/0/car	81.63	73.51	70.41	47.60	68.37	40.28	**98.29**	**89.54**	**98.29**	**76.98**	**98.63**	**68.84**
11/0/car	72.65	74.78	61.66	50.74	52.58	47.35	**97.85**	**87.71**	**97.85**	**84.42**	**98.12**	**74.04**
11/35/car	53.17	65.25	19.05	31.95	6.35	26.02	**77.34**	**84.09**	**71.09**	**84.74**	**76.56**	**74.43**
18/2/car	86.36	74.81	67.05	45.47	62.12	34.80	**93.18**	**89.84**	**93.18**	**80.92**	**93.18**	**74.46**
18/3/car	53.33	70.94	21.75	41.45	16.84	35.80	**85.96**	**86.20**	**85.61**	**77.06**	**85.96**	**64.50**
19/63/car	35.26	63.50	29.48	45.69	26.48	33.89	**54.91**	**90.89**	**53.76**	**84.65**	**55.49**	**77.93**
19/72/car	**29.11**	62.59	**29.43**	55.48	**29.43**	39.81	12.34	**75.67**	12.03	**73.30**	12.03	**61.74**
20/0/car	63.68	78.54	43.78	45.00	31.84	46.15	**85.00**	**90.33**	**85.50**	**74.98**	**86.00**	**65.06**
20/12/car	42.77	76.77	37.64	49.29	36.23	40.81	**91.91**	**88.24**	**90.05**	**73.67**	**91.76**	**62.03**
20/122/car	34.90	78.76	34.51	48.05	29.02	44.43	**90.98**	**83.21**	**77.25**	**72.15**	**91.37**	**60.27**
mean	52.65	70.96	39.05	47.01	34.10	39.31	**80.48**	**86.35**	**78.94**	**77.95**	**80.87**	**67.97**
std	**19.01**	6.20	**17.82**	6.13	28.30	6.36	23.49	**4.09**	23.47	**4.40**	**23.69**	**5.63**

Bold values indicate the best performance for each metric in the corresponding row.

**Table 3:** Ablation study on ego pose estimation.

seq	No Keypoint–Object Layer		No GNN		No Gating Layer		No Soft Assignment		KOM-SLAM	
	RPE_*t*_ (m/f)	RPE_*R*_ (°/f)	RPE_*t*_ (m/f)	RPE_*R*_ (°/f)	RPE_*t*_ (m/f)	RPE_*R*_ (°/f)	RPE_*t*_ (m/f)	RPE_*R*_ (°/f)	RPE_*t*_ (m/f)	RPE_*R*_ (°/f)
0000	**0.04**	**0.07**	**0.04**	0.10	**0.04**	0.08	**0.04**	0.09	**0.04**	**0.07**
0001	**0.04**	0.15	**0.04**	**0.01**	**0.04**	0.09	**0.04**	0.02	**0.04**	0.03
0002	**0.03**	**0.00**	**0.03**	0.06	**0.03**	0.03	**0.03**	0.01	**0.03**	**0.00**
0003	0.06	**0.02**	0.06	0.04	0.06	0.06	0.06	0.03	**0.05**	**0.02**
0004	**0.06**	0.08	**0.06**	**0.07**	0.07	**0.07**	0.07	0.10	**0.06**	**0.07**
0005	**0.05**	0.10	**0.05**	**0.01**	**0.05**	0.03	**0.05**	0.14	**0.05**	**0.01**
0006	**0.01**	0.06	**0.01**	0.06	**0.01**	**0.01**	0.02	0.11	**0.01**	**0.01**
0007	**0.04**	0.10	**0.04**	0.18	**0.04**	0.29	0.05	0.09	**0.04**	**0.03**
0008	**0.07**	**0.03**	**0.07**	0.07	**0.07**	0.05	**0.07**	0.04	**0.07**	**0.03**
0009	**0.04**	0.03	**0.04**	0.05	**0.04**	0.03	**0.04**	0.05	**0.04**	**0.02**
0010	**0.06**	0.15	**0.06**	0.04	**0.06**	0.03	**0.06**	0.05	**0.06**	**0.02**
0011	**0.03**	0.15	**0.03**	0.12	**0.03**	**0.03**	0.04	0.20	**0.03**	0.04
0013	**0.03**	0.08	**0.03**	0.05	**0.03**	0.04	**0.03**	0.06	**0.03**	**0.02**
0014	**0.03**	0.09	**0.03**	0.09	**0.03**	0.07	**0.03**	0.10	**0.03**	**0.05**
0018	**0.04**	0.11	**0.04**	0.03	**0.04**	0.03	**0.04**	0.03	**0.04**	**0.00**
0019	**0.03**	0.17	**0.03**	0.11	**0.03**	0.11	**0.03**	0.05	**0.03**	**0.02**
0020	**0.04**	0.03	**0.04**	**0.00**	**0.04**	0.02	**0.04**	0.01	**0.04**	0.01
mean	**0.041**	0.084	**0.041**	0.064	0.042	0.062	0.043	0.069	**0.041**	**0.028**
std	0.015	0.050	0.015	0.044	0.015	0.062	**0.014**	0.049	**0.014**	**0.020**

Bold values indicate the best performance for each metric in the corresponding row.

**Table 4:** Ablation study on object tracking.

seq	Naive Approach		No GNN		No Keypoint–Object Layer		KOM-SLAM	
	MOTA	IDF1	MOTA	IDF1	MOTA	IDF1	MOTA	IDF1
0000	**1.00**	0.97	0.99	0.98	**1.00**	**1.00**	**1.00**	**1.00**
0001	0.94	0.91	0.96	0.91	**0.99**	0.94	**0.99**	**0.96**
0002	0.91	0.90	0.92	0.68	0.98	0.88	**0.99**	**0.91**
0003	0.59	0.59	0.97	0.89	0.97	0.95	**1.00**	**1.00**
0004	0.70	0.70	0.99	0.97	0.98	0.98	**1.00**	**0.99**
0005	0.77	0.74	0.97	0.92	0.97	0.95	**1.00**	**0.99**
0006	0.25	0.25	**0.99**	**0.95**	**0.99**	0.85	**0.99**	0.87
0007	0.95	0.95	0.99	0.96	**1.00**	0.99	**1.00**	**1.00**
0008	0.32	0.32	0.97	0.80	0.85	0.71	**1.00**	**0.92**
0009	0.83	0.80	0.97	0.87	0.98	0.93	**0.99**	**0.94**
0010	0.72	0.70	0.94	0.88	0.96	0.96	**1.00**	**1.00**
0011	0.87	0.87	0.96	0.83	0.98	0.93	**0.99**	**0.95**
0013	**1.00**	**0.99**	0.95	0.85	0.91	0.84	0.95	0.92
0014	0.89	0.88	0.89	0.82	0.90	0.85	**0.92**	**0.88**
0018	0.73	0.69	**0.99**	0.81	0.96	0.87	**0.99**	**0.88**
0019	**0.99**	**0.99**	0.95	0.75	0.96	0.75	0.97	0.82
0020	0.92	0.92	0.98	0.92	**0.99**	0.91	**0.99**	**0.97**
mean	0.79	0.77	0.96	0.88	0.96	0.90	**0.99**	**0.94**
std	0.22	0.21	0.027	0.077	0.039	0.079	**0.021**	**0.054**

Bold values indicate the best performance for each metric in the corresponding row.

**Table 5:** Ablation study on data density and time gap.

seq	Sparser Keypoints		Every 2 Frames		KOM-SLAM	
	RPE_*t*_ (m/f)	RPE_*R*_ (°/f)	RPE_*t*_ (m/f)	RPE_*R*_ (°/f)	RPE_*t*_ (m/f)	RPE_*R*_ (°/f)
0000	**0.04**	0.08	0.07	0.28	**0.04**	**0.07**
0001	**0.04**	**0.02**	0.08	0.14	**0.04**	0.03
0002	**0.03**	0.06	0.04	0.05	**0.03**	**0.00**
0003	0.06	0.13	0.09	0.12	**0.05**	**0.02**
0004	0.07	0.08	0.12	0.31	**0.06**	**0.07**
0005	**0.05**	**0.01**	0.06	**0.01**	**0.05**	**0.01**
0006	0.02	**0.01**	0.04	0.10	**0.01**	**0.01**
0007	0.05	0.08	0.14	0.33	**0.04**	**0.03**
0008	**0.07**	0.05	0.09	0.13	**0.07**	**0.03**
0009	0.05	0.05	0.10	0.25	**0.04**	**0.02**
0010	**0.06**	0.09	0.08	0.09	**0.06**	**0.02**
0011	**0.03**	**0.01**	0.05	0.13	**0.03**	0.04
0013	**0.03**	0.05	0.05	0.19	**0.03**	**0.02**
0014	**0.03**	0.09	0.06	0.14	**0.03**	**0.05**
0018	**0.04**	**0.00**	0.08	0.04	**0.04**	**0.00**
0019	**0.03**	0.07	0.06	0.07	**0.03**	**0.02**
0020	**0.04**	0.07	0.09	0.06	**0.04**	**0.01**
mean	0.043	0.055	0.076	0.14	**0.041**	**0.028**
std	0.015	0.034	0.026	0.094	**0.014**	**0.020**

Bold values indicate the best performance for each metric in the corresponding row.

## Data Availability

The original data presented in the study are openly available at https://www.cvlibs.net/datasets/kitti/eval_tracking.php (accessed on 8 March 2025).
